# The Puf family of RNA-binding proteins in plants: phylogeny, structural modeling, activity and subcellular localization

**DOI:** 10.1186/1471-2229-10-44

**Published:** 2010-03-09

**Authors:** Patrick PC Tam, Isabelle H Barrette-Ng, Dawn M Simon, Michael WC Tam, Amanda L Ang, Douglas G Muench

**Affiliations:** 1 Department of Biological Sciences, University of Calgary, 2500 University Dr NW Calgary, AB T2N 1N4, Canada; 2 Department of Biology, University of Nebraska at Kearney, 905 W 25th Street, Kearney, NE 68849, USA

## Abstract

**Background:**

Puf proteins have important roles in controlling gene expression at the post-transcriptional level by promoting RNA decay and repressing translation. The Pumilio homology domain (PUM-HD) is a conserved region within Puf proteins that binds to RNA with sequence specificity. Although Puf proteins have been well characterized in animal and fungal systems, little is known about the structural and functional characteristics of Puf-like proteins in plants.

**Results:**

The Arabidopsis and rice genomes code for 26 and 19 Puf-like proteins, respectively, each possessing eight or fewer Puf repeats in their PUM-HD. Key amino acids in the PUM-HD of several of these proteins are conserved with those of animal and fungal homologs, whereas other plant Puf proteins demonstrate extensive variability in these amino acids. Three-dimensional modeling revealed that the predicted structure of this domain in plant Puf proteins provides a suitable surface for binding RNA. Electrophoretic gel mobility shift experiments showed that the Arabidopsis AtPum2 PUM-HD binds with high affinity to BoxB of the Drosophila Nanos Response Element I (NRE1) RNA, whereas a point mutation in the core of the NRE1 resulted in a significant reduction in binding affinity. Transient expression of several of the Arabidopsis Puf proteins as fluorescent protein fusions revealed a dynamic, punctate cytoplasmic pattern of localization for most of these proteins. The presence of predicted nuclear export signals and accumulation of AtPuf proteins in the nucleus after treatment of cells with leptomycin B demonstrated that shuttling of these proteins between the cytosol and nucleus is common among these proteins. In addition to the cytoplasmically enriched AtPum proteins, two AtPum proteins showed nuclear targeting with enrichment in the nucleolus.

**Conclusions:**

The Puf family of RNA-binding proteins in plants consists of a greater number of members than any other model species studied to date. This, along with the amino acid variability observed within their PUM-HDs, suggests that these proteins may be involved in a wide range of post-transcriptional regulatory events that are important in providing plants with the ability to respond rapidly to changes in environmental conditions and throughout development.

## Background

Post-transcriptional control of gene expression functions to regulate protein synthesis in a spatial and temporal manner, and involves the activity of an extensive array of RNA-binding proteins. Throughout the lifetime of an mRNA, a dynamic association exists between mRNAs and RNA-binding proteins. These interactions are important in mediating mRNA maturation events such as splicing, capping, polyadenylation and export from the nucleus [1,2]. RNA-binding proteins also contribute to post-transcriptional regulatory events in the cytoplasm, such as mRNA localization, mRNA stability and decay, and translation. One group of RNA-binding proteins that are important regulators of cytoplasmic post-transcriptional control is the Puf family of proteins. Puf proteins have extensive structural conservation within their RNA binding domain and regulate a range of biological processes, including developmental patterning, stem cell control, and neuron function [3].

The founding members of the Puf family of proteins are Pumilio in Drosophila and *fem-3 *binding factor (FBF) in *C. elegans *[4,5]. Puf protein diversity extends across kingdoms, as mammalian, fungal, protozoan and plant homologs have been identified [6-8]. The number of Puf gene copies in each model organism is variable. For example, the Drosophila, human, yeast, and *C. elegans *genomes encode one, two, six and eleven Puf genes, respectively [9]. Puf proteins are generally known to bind directly to sequence elements located within the 3' untranslated region (UTR) of their target mRNAs. Once bound, they interact with other proteins to inhibit translation or trigger mRNA decay. For instance, Drosophila Pumilio represses the translation of *hunchback *(*hb*) mRNA in early embryo development through deadenylation dependent and independent mechanisms [10]. Pumilio binds to a pair of 32 nucleotide Nanos Response Elements (NRE1 and NRE2) located within the 3'UTR of the *hunchback *mRNA. Each NRE contains two core elements (Box A and Box B), each of which interacts with one Pumilio protein in a cooperative manner [11]. This interaction provides a platform for the recruitment of Nanos (Nos) and Brain Tumor (Brat) proteins to repress the translation of *hunchback *mRNA in the posterior region of the embryo.

The RNA binding domain of Puf proteins (the Pumilio Homology Domain, PUM-HD) forms a crescent-shaped structure that usually contains eight imperfect tandem Puf repeats each consisting of approximately 36 amino acids [6,7]. Each Puf repeat is organized into three α-helices, the second of which provides a binding interface with the target RNA. Within each Puf repeat, three conserved amino acid side chains are typically responsible for modular binding of the repeat to a single RNA base using hydrogen bonds, van der Waals, and base stacking interactions [12]. Puf proteins often bind target transcripts that contain a conserved UGUR (where R represents a purine) tetranucleotide motif flanked downstream by an AU-rich sequence of four nucleotides. The modular binding of each Puf repeat to an RNA base is predictable based on the combination of specific amino acids that contact the Watson-Crick edge of the base [12-14]. This interaction, however, demonstrates considerable complexity and adaptability, as a wide range of RNA sequences are recognized by each Puf protein. For example, RNA-immunoprecipitation profiling studies have shown that individual Puf proteins can bind to hundreds of unique transcripts *in vivo *[15-18]. This suggests that that this family of proteins has important roles in regulating the stability and translation of numerous mRNA targets across a broad range of organisms. These and other studies have shown that Puf proteins can recognize RNA sequences that extend beyond the canonical eight nucleotide length, and can bind to non-cognate sequences [14,19-21]. The identification of mRNA targets of individual Puf proteins has revealed that Puf proteins typically bind to subsets of mRNAs that are functionally or cytotopically related and located within macromolecular complexes. Thus, related groups of mRNAs may be coordinately regulated as 'post-transcriptional operons' or 'RNA regulons' [15,16,22,23]. For example, yeast Puf3p binds to motifs located in the 3'UTR of numerous mRNAs that encode mitochondrial proteins and regulates the stability, transport and translation of these transcripts [24]. The RNA regulon model predicts that environmental cues result in a dynamic remodeling of RNP complexes to co-regulate mRNAs in a combinatorial manner to serve various functional roles within the cell [22].

Plant Puf proteins have been described only briefly in the literature, in the form of limited phylogenetic analyses [9,16,25,26], and recently with the identification of putative mRNA targets of Arabidopsis Puf proteins [27]. Here, we discuss the evolutionary relationships of the complete set of Puf proteins from the dicotyledonous plant *Arabidopsis thaliana *(Arabidopsis) and the monocotyledonous plant *Oryza sativa *(rice), as well as members from a moss and algal species. We also describe three-dimensional structural modeling, and biochemical and cellular characteristics of selected members of this protein family. This work demonstrates that the plant PUM-HD adopts the typical crescent shaped structure that is characteristic of this domain in other organisms, and that it possesses sequence specific RNA binding activity *in vitro*. We provide evidence these plant Puf proteins are packaged into common cytoplasmic particles that presumably have an evolutionary conserved role in the post-transcriptional control of a vast array of mRNA targets.

## Results

### Identification and comparative analysis of plant Puf proteins

**Figure 1:**
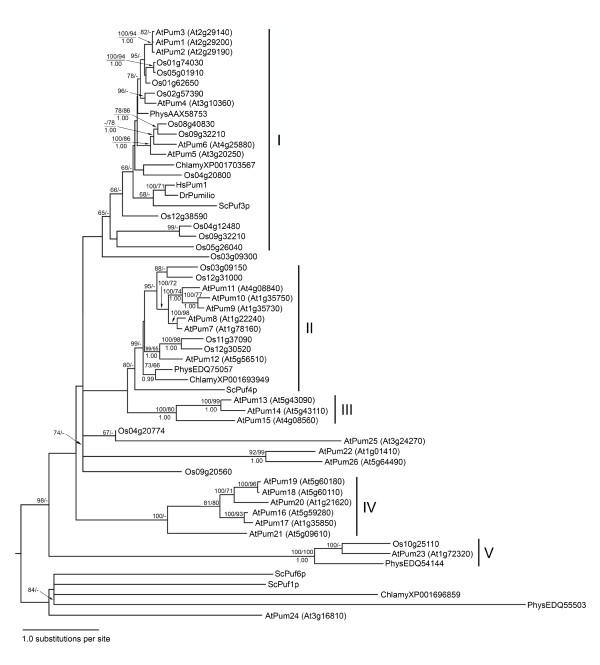
**A maximum likelihood phylogenetic tree of the PUM-HDs of Arabidopsis, rice and other plant and non-plant species**. The analysis is based on the deduced amino acid sequence of the PUM-HD domain from each predicted Puf gene. The tree includes all members from Arabidopsis and rice, and representative members from *Physcomitrella patens *(Phys), *Chlamydomonas reinhardii *(Chlamy), *Saccharomyces cerevisiae *(Sc), as well as Drosophila Pumilio (DrPumilio) and human Pum1 (HsPum1). The Arabidopsis genes are referred by their designated Pum gene number (i.e., AtPumxx) that were reported by the National Center for Biotechnology Information (NCBI), as well as their gene locus name (Atxxxxxxx). The rice clones are identified by their gene locus name only (Osxxxxxxxx), as standardized Pum gene designations have not yet been established. Maximum likelihood bootstrap values (>65%) are shown above the nodes (PhyML/RaxML), and Bayesian posterior probability values (>0.95) are shown below the nodes. The bar at the bottom of the figure indicates the number of substitutions per site. The tree is rooted at its midpoint and, thus, its rooting should be interpreted as an hypothesis.

BLASTp and tBLASTn searches of the Arabidopsis and rice genome databases were conducted using the Drosophila Pumilio PUM-HD amino acid sequence (residues 1093 to 1427) as the query sequence. This search revealed that both the Arabidopsis and rice genomes encode strikingly large Puf gene families that include 26 and 19 putative members, respectively. A phylogenetic tree of the predicted Arabidopsis and rice Puf proteins was constructed based on the deduced amino acid sequence of their PUM-HD coding sequence (Figure 1). Also included in the phylogenetic tree were representative Puf sequences from the moss *Physcomitrella patens*, the green alga *Chlamydomonas reinhardtii*, and the yeast *Saccharomyces cerevisiae*, as well as Drosophila Pumilio and human Pum1.

The phylogentic tree identified several sub-families of proteins that were assigned into groups based on monophyly (Figure 1). Group I was the most extensive of all groups, and contained at least one Puf member from each of the species that were included in this analysis. This group corresponds to the 'Pumilio cluster' of proteins that was categorized previously [9]. Group II contained plant, algal, and yeast proteins, whereas Groups III, IV, and V contained plant members only. A number of proteins are more divergent, and do not appear to belong to any of the major branches that were identified in this analysis (Figure 1). Some Arabidopsis and rice Puf genes appear to be orthologs (e.g., AtPum4 and Os02g57390, and AtPum23 and Os10g25110) as they demonstrate a high degree of sequence conservation in the PUM-HD. Additionally, two *Chlamydomonas *proteins (XP001703567 and XP001693949) also appear to be orthologs with plant Puf proteins. Gene expansion through tandem duplication is also evident from this analysis. AtPum 1, 2, and 3 (Group I) are clustered in one region of chromosome 2, and other tandemly located genes are also evident (i.e., AtPum 9 and 10, AtPum 13 and 14, and AtPum 18 and 19).

**Figure 2:**
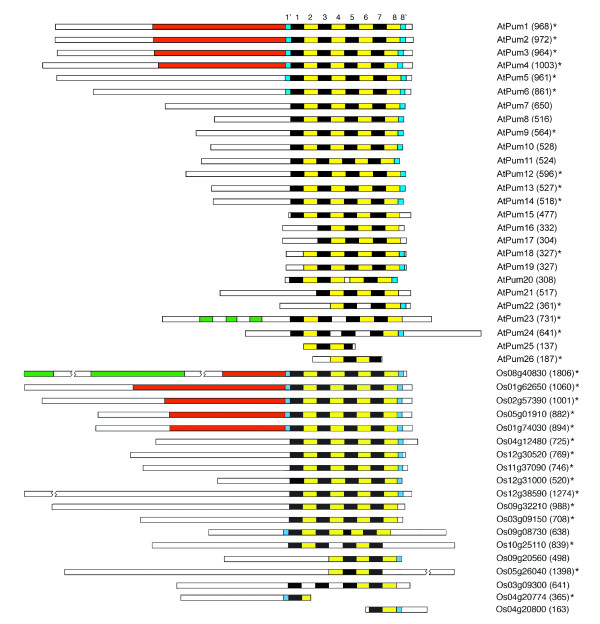
**Schematic line diagram comparing the primary structure of Puf proteins in Arabidopsis and rice**. The numbered Puf repeats in the PUM-HD of each protein are indicated (alternating black and yellow strips), and the 1' and 8' pseudorepeats are also identified (blue). A conserved nucleic acid binding protein domain (NABP) is present in several Arabidopsis and rice PUM-HDs (red). Three additional Puf repeats were identified outside of the PUM-HD in AtPum23 (green). Two versions of the 'domain of unknown function' (DUF) were identified in Os08g40830 (green). The length of each protein is indicated in parentheses. Sequences that are supported by cDNA sequences are identified (*). The AtPum13 and AtPum22 cDNAs were amplified and sequenced independently (PPC Tam and DG Muench, unpublished observations).

Greater than half of the Arabidopsis (15/26) and rice (13/19) Puf proteins possess eight imperfect tandem Puf repeats (Figure 2). This is consistent with the number of Puf repeats present in most non-plant Puf proteins, although examples of functional Puf proteins with fewer than eight repeats have been identified [15]. The remaining Arabidopsis and rice Puf proteins lack one or more of these repeats, with some possessing only two or three obvious repeats. A number of core residues are uniquely conserved within each of the eight PUF repeats, thereby allowing us to determine the identity of each repeat and whether a specific repeat is absent or truncated. Crystallographic studies have demonstrated that the eight tandem Puf repeats of the human PUM-HD are flanked by two imperfect pseudorepeats (1' and 8') [7]. Regions resembling these pseudorepeats are present in several of the Arabidopsis and rice proteins (Figure 2). Puf proteins from other species often contain large regions of low complexity [15]. Although isolated, short regions of repeated amino acids are observed in some Arabidopsis and rice Puf proteins, extensive stretches of low complexity sequence are not observed in these proteins. The tandemly positioned rice open reading frames (ORFs), Os04g20774 and Os04g20800, possess amino and carboxyl ends of the PUM-HD, respectively (Figure 2). Analysis of the genomic DNA region that separates the two sequences identified a transposon that likely inserted within a full-length PUM-HD from the ancestral Puf protein. Interestingly, there is cDNA support for Os04g20774, suggesting that the encoded protein is functional. Although Os04g20774 and Os04g20800 are placed in different positions in the phylogenetic tree (Figure 1), Os04g20774 likely belongs, by association, with Os04g20800 in Group I. Placing Os04g20774 in clade with AtPum25 is likely coincidental, as there is little conservation between these two sequences.

Those Arabidopsis and rice genes that were not supported by cDNA sequences (Figure 2) were analyzed more extensively in an attempt to validate their predicted ORFs. The presence of many closely related members within each of the Arabidopsis and rice Puf families allowed for sequence comparisons to provide a more confident assignment of ORFs. Notably, the ORFs of AtPum15 and AtPum17 that were listed in the database appear to have incorrectly predicted introns. In the case of AtPum15, this resulted in the merger of an ORF encoding a self-incompatibility protein with that of AtPum15. An incorrectly predicted intron in AtPum17 was likely the result of a sequencing error. This predicted intron contained sequence that was almost identical to sequence within the ORF of the intronless gene AtPum16, a close relative of AtPum17. Based on this information, the primary structure line diagrams have been modified, with the removal of the self-incompatibility ORF from AtPum15, and the intron from AtPum17 (Figure 2).

**Figure 3:**
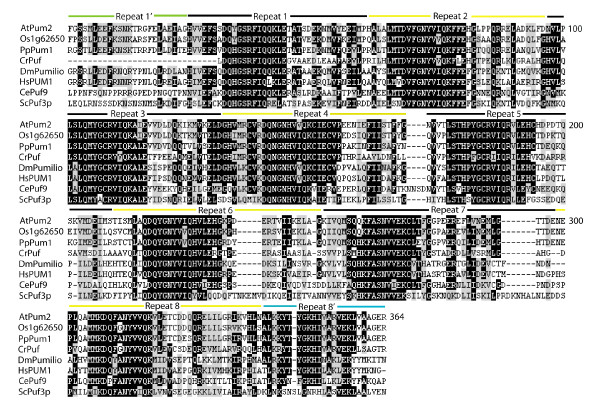
**Amino acid sequence alignment of the PUM-HD encoded by Puf genes in various organisms**. *Arabidopsis thaliana *(AtPum2); *Oryza sativa *(Os01g62650); *Physcomitrella patens *(PpPum1, AAX58753); *Chlamydomonas reinhardii *(CrPuf, XP001703567); *Drosophila melanogaste*r (DmPumilio); *Homo sapiens *(HsPUM1);*Caenorhabditis elegan*s (CePuf9); and *Saccharomyces cerevisiae *(ScPuf3p). Identical amino acids are marked in black and similar residues are marked in gray.

The Arabidopsis PUM-HD with the highest amino acid sequence similarity to the human Pum1 PUM-HD is AtPum2, sharing 54% amino acid identity within this domain. The rice Puf protein with the highest amino acid sequence identity to AtPum2 is Os01g62650, possessing 49% amino acid identity throughout the entire protein and 84% identity within the PUM-HD. The AtPum2 and Os01g62650 PUM-HDs were included in an amino acid sequence alignment with PUM-HDs from other plant and non-plant species, and this alignment demonstrated that extensive sequence conservation exists in each of the Puf repeats (Figure 3). A comprehensive amino acid alignment of PUM-HDs comparing the Arabidopsis and rice PUM-HDs with all of the *P. patens*, *C. reinhardtii*, *S. cerevisiae*, human and Drosophila PUM-HDs demonstrated that the core of each repeat has a high degree of amino acid conservation across species (Additional file 1). The *P. patens *genome contains 11 Puf-like genes, whereas four Puf-like genes are present in the *C. reinhardtii *genome.

**Figure 4:**
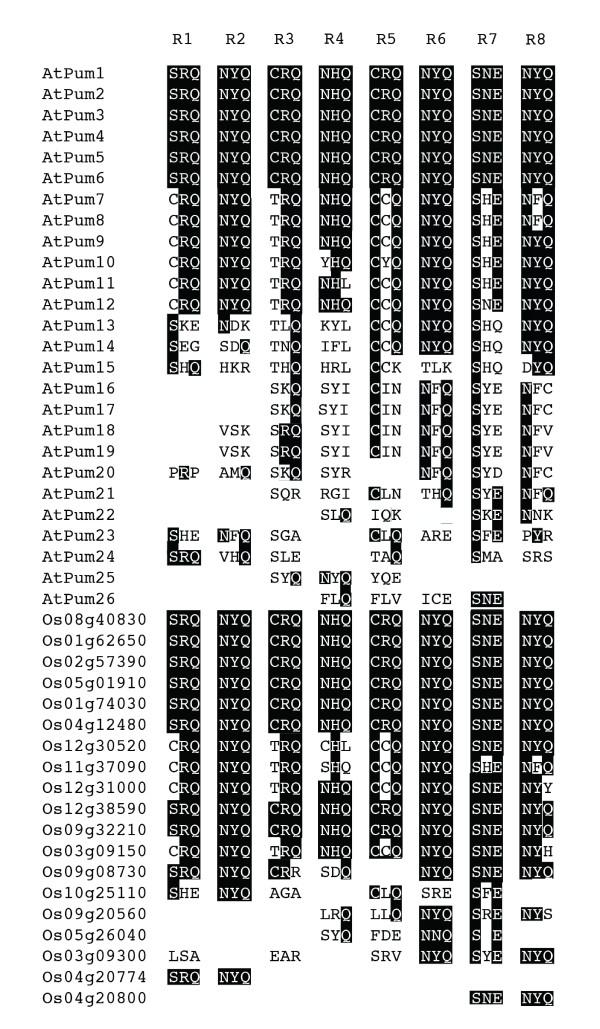
**Alignment of amino acids in the PUM-HD that are predicted to interact with RNA bases**. Sequence alignment of amino acid triplets at positions 12, 13 and 16 in each Puf repeat (R1 to R8) from the Arabidopsis and rice Puf proteins. Black shading identifies amino acids that are identical to the human Pum1 protein.

Crystallographic analysis of Puf proteins from other species has determined that the amino acids at positions 12, 13 and 16 within each Puf repeat provide the binding interface with RNA bases using hydrogen bonds, van der Waals, or stacking interactions [13]. Surprisingly, alignment of these triplet amino acids in Puf repeats from the Arabidopsis and rice PUM-HDs demonstrated that there is complete conservation in some members and extensive variability in others (Figure 4). The amino acids at positions 12, 13 and 16 from AtPum1 through AtPum6 are conserved with the corresponding triplets in human Pum1 and Drosophila Pumilio (Figure 3, 4; [6,12]). However, AtPum7 through AtPum12 possess a single amino acid substitution in several of these amino acid triplets, and AtPum13 through AtPum26 show extensive variability and are less easily predictable (Figure 4). The rice PUM-HDs showed less variability in these triplets, although uncommon triplet combinations were also evident. In some Arabidopsis and rice PUM-HDs, amino acid substitutions in one Puf repeat resulted in a triplet composition that is identical to that observed in a different Puf repeat (Figure 4). For instance, repeat 1 in several of the Arabidopsis and rice proteins possesses a cysteine at position 12 (CRQ), resulting in an amino acid triplet that matches that of Puf repeats 3 and 5 in the conserved proteins. Interestingly, this CRQ triplet is also found in repeat 1 in some fungal and protozoan Puf proteins [16]. Several examples of unconventional triplets are present in the Arabidopsis and rice Puf repeats (Figure 4), some of which are present in Puf repeats of other species as well (Additional file 1) [16,28].

The regions of the Arabidopsis and rice Puf proteins that lie outside of the PUM-HD are variable in primary sequence and length (Figure 2, Additional file 2). These variable sequences are typically amino-terminal extensions of each protein, although carboxyl-terminal extensions of variable length are also present in several proteins. A Pfam search http://pfam.sanger.ac.uk/ of the polypeptide regions lying outside of the PUM-HD was performed in an attempt to identify significantly conserved domains that are present within the variable regions of the Arabidopsis and rice Puf proteins. AtPum23 is the only Arabidopsis or rice Puf protein that possesses Puf repeat sequences that reside outside of the conserved PUM-HD region (Figure 2). Additionally, the amino-terminal region of several related Arabidopsis and rice proteins within Group I (Figure 1) possess a motif that resembles a Nucleic Acid Binding Protein domain (NABP, pfam07990; [29])(Figure 2). Finally, the rice protein Os08g40830 possesses two regions in its amino terminal extension that are similar to versions of a 'domain of unknown function' (DUF, pfam04782, pfam04783)(Figure 2), a region found in some leucine zipper proteins [30].

To gain insight into the expression pattern of the Arabidopsis Puf genes in different tissues and in response to various environmental stimuli, the transcription profiles for these genes were extracted from the microarray database [31,32]. Some overlap exists in the tissues/organs that exhibit maximal expression between Arabidopsis Puf genes, particularly those genes that are closely related (Table 1). Each of the Puf genes showed a significant change in expression pattern in response to at least one abiotic or biotic stimulus. Extensive variability exists in the type of response to these stimuli, even between genes that are closely related (Table 1).

**Table 1:** AtPum transcript expression based on available public database information.

Gene	Organ/tissue with highest expression	Stimulus resulting in significant changes in transcript level
Pum 1 (At2g29200)	Hypocotyl - xylem	Nutrient - cesium
Pum 2 (At2g29190)	Hypocotyl - xylem	Heat, 2,4-dichlorophenoxyacetic acid
Pum 3 (At2g29140)	Hypocotyl - xylem	Nutrient - cesium
Pum 4 (At3g10360)	Stamen - pollen	Nematode (*H. schachtii*)
Pum 5 (At3g20250)	Hypocotyl - xylem	Light - extended night, Osmotic stress
Pum 6 (At4g25880)	Hypocotyl - xylem	*A. tumefaciens *- inoculated with cabbage leaf curl virus
Pum 7 (At1g78160)	Flower - stamen	Iron deficiency
Pum 8 (At1g22240)	Endosperm - micropylar endosperm	Exposure to unfiltered UV-B light
Pum 9 (At1g35730)	Hypocotyl - xylem	Drought
Pum 10 (At1g35750)	Hypocotyl - xylem	Exposure to unfiltered UV-B light
Pum 11 (At4g08840)	Root - lateral root	2,4-dichlorophenoxyacetic acid
Pum 12 (At5g56510)	Seed coat - chalazal seed coat	*A. tumefaciens*, Nematode, Cycloheximide, Drought
Pum 13 (At5g43090)	Vegetative shoot apex	Salt stress
Pum 14 (At5g43110)	Endosperm - micropylar endosperm	Dark, Iron deficiency
Pum 15 (At4g08560)	Endosperm - chalazal endosperm	Nitrate deficiency, Sucrose
Pum 16 (At5g59280)	Flower - pollen	ABA
Pum 17 (At1g35850)	Mature pollen grain	Sucrose deficiency
Pum 18 (At5g60110)	Endosperm - peripheral endosperm	Brassinolide, H_3_BO_3_
Pum 19 (At5g60180)	Young expanding leaf (Stage 4)	Osmotic stress
Pum 20 (At1g21620)	Young expanding leaf (Stage 4)	Osmotic stress
Pum 21 (At5g09610)	Senescing leaf (35 days old)	Salt stress
Pum 22 (At1g01410)	Root- stele	Hypoxia
Pum 23 (At1g72320)	Imbibed seed	ABA
Pum 24 (At3g16810)	Root - root tip	Glucose
Pum 25 (At3g24270)	Root - lateral root cap	Drought
Pum 26 (At5g64490)	Imbibed seed	*A. tumefaciens*

### Three-dimensional models of plant PUM-HDs

A homology modeling approach was used to gain insight into whether plant PUM-HDs adopt the typical crescent shaped three-dimensional structure similar to that of the PUM-HDs from human, Drosophila and yeast Puf proteins. The three-dimensional models of the AtPum2 and Os01g62650 PUM-HDs bound to BoxB of the *hunchback *mRNA NRE1 were constructed using the crystal structure of the PUM-HD from human Pum1 bound to the NRE1 RNA (PDB: 1M8X; [12]) as a template for homology modeling. This structure was determined at 2.2 Å resolution and provides the most reliable template currently available for modeling the nature of protein:RNA interactions from plant PUM-HDs. Notably, only interactions between Puf repeats 2 to 8 and the bound RNA could be modeled, since the RNA templates for the complexes determined at high resolution only included residues 1 to 9 of Box B from NRE1 (PDB: 1M8X and 1M8W; [12]).

**Figure 5:**
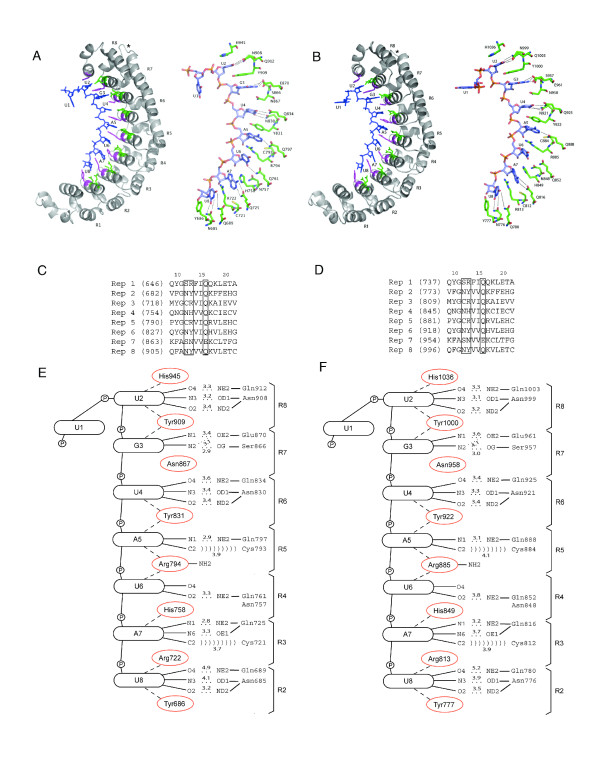
**Models of the plant PUM-HD bound to RNA**. (A, B) Ribbon (left) and stick (right) models of the PUM-HDs of AtPUM2 (A) and Os01g62650 (B) bound to the RNA bases of Box 2 of the NRE (UUGUAUAU) that interact with Puf repeats 2 to 8. The RNA is shown as a ball-and-stick model. In the ribbon diagrams, the amino acid side chains that interact with the Watson-Crick edge of each base are shown in green, and those that provide potential stacking interactions are colored magenta. In the stick models, only the amino acid side chains that contact RNA bases are shown. The extended loop between repeat 7 and 8 is identified (*). (C, D) Sequence alignment of residues in helix 2 of repeats 1-8 that provide putative RNA contact sites on the concave surface of the PUM-HD of AtPum2 (C) and Os01g62650 (D). Numbers above the sequences represent the position of each amino acid each Puf repeat. Numbers in brackets refer to the position of the first amino acid in the complete AtPum2 and Os01g62650 polypeptide sequence. Boxes surround the amino acid residues at positions 12, 13 and 16. (E, F) Schematic diagram showing the protein:RNA contacts in the models of the AtPum2 (E) and Os01g62650 (F) PUM-HDs bound to the NRE1. Dotted lines indicate potential hydrogen bonds, dashed lines indicate potential stacking interactions, and ')))))' indicates potential van der Waals interactions. Distances between atoms indicated on the lines are indicated in Ångstroms.

The homology models of the AtPum2 and Os01g62650 PUM-HD bound to the NRE1 indicate that plant PUM-HDs can form interactions with RNA in a manner similar to that observed in the human PUM-HD:RNA complexes (Figure 5A, B, Additional file 3; [12]). The conserved amino acid triplets at position 12, 13, and 16 of each repeat in AtPum2 and Os01g62650 (Figure 4, Figure 5C, D) form interactions with RNA bases in the modeled structure (Figure 5A, B, E, F). Most of the hydrogen bonds and van der Waals contacts formed by amino acids at positions 12 and 16 in the human PUM-HD:RNA crystal structures [12] are also observed in the models of the plant PUM-HD:RNA complexes (Figure 5E, F). The stacking interactions between residues at position 13 and adjacent bases are also conserved. In addition to similarities in the structures of the Puf repeats, the homology models also indicate that a region lying between the seventh and eighth Puf repeats can form an extended loop structure on the convex surface of the domain (Figure 5A, B), similar to that observed in the human and Drosophila PUM-HD proteins. In Drosophila, this loop interacts with the translational co-repressors Nos and Brat [6,33].

**Figure 6:**
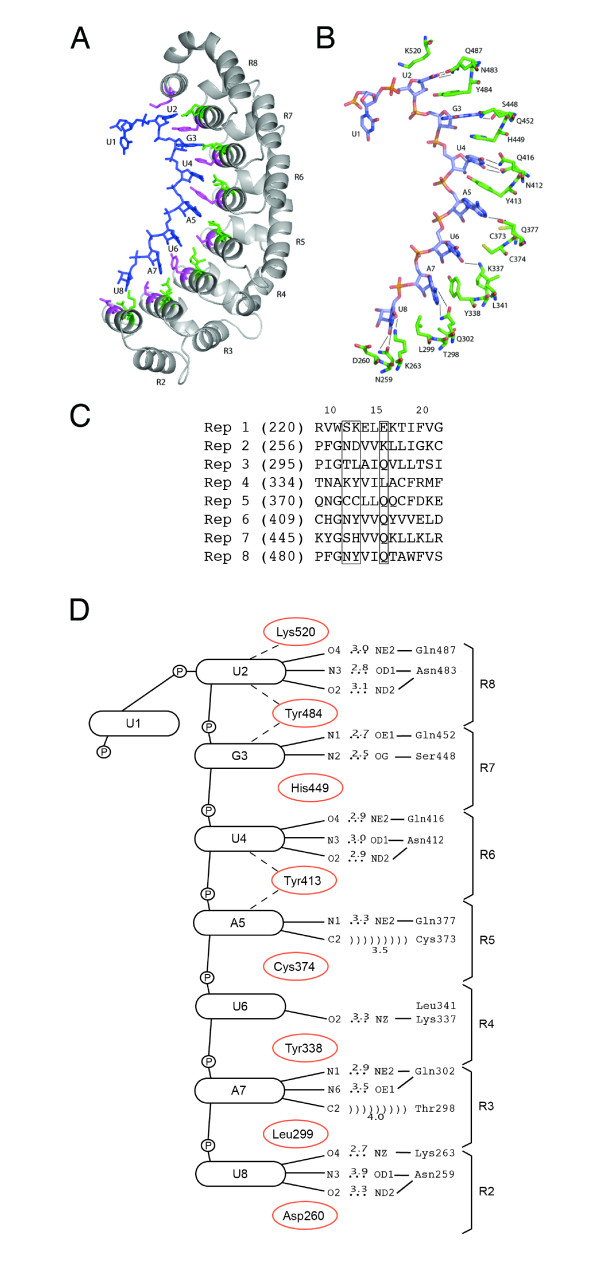
**Models of the AtPum13 PUM-HD bound to RNA**. Ribbon (A) and stick (B) models of the PUM-HD of AtPUM13 bound to the core nucleotides of Box 2 of the NRE1 (UUGUAUAU). (C) Sequence alignment of residues in helix 2 of repeats 1-8 that provide putative RNA contact sites on the concave surface of the PUM-HD. (D) Schematic diagram showing the protein:RNA contacts in the model of the AtPum13 PUM-HD. Legend details are described in Figure 5.

A similar approach was used to model the structure of the PUM-HD of AtPum13, a Puf protein that varies significantly in the identity of Puf repeat amino acid residues at positions 12, 13 and 16 (Figure 4). The homology model for the AtPum13 PUM-HD:RNA complex indicates that interactions between Puf repeats 6, 7 and 8 with the highly conserved UGU sequence at the centre of Box B are conserved in AtPum2 and Os01g62650 (compare Figure 5 with Figure 6). However, the model also shows that the remaining AtPum13 Puf repeats fail to form many of the stacking interactions and hydrogen bond interactions that are observed in AtPum2 and Os01g62650. As a result, we predict that the binding affinity of AtPum13 for the NRE1 is lower than that of AtPum2, and AtPum13 may prefer RNA targets that are different from the NRE1 outside of the UGU core. It is also interesting to note that the AtPum13 model reveals the presence of extended loops on the convex surface of the protein between Puf repeats 2 and 3, as well as repeats 3 and 4 (Figure 6).

### AtPum2 PUM-HD binds with specificity to the hunchback NRE1

**Figure 7:**
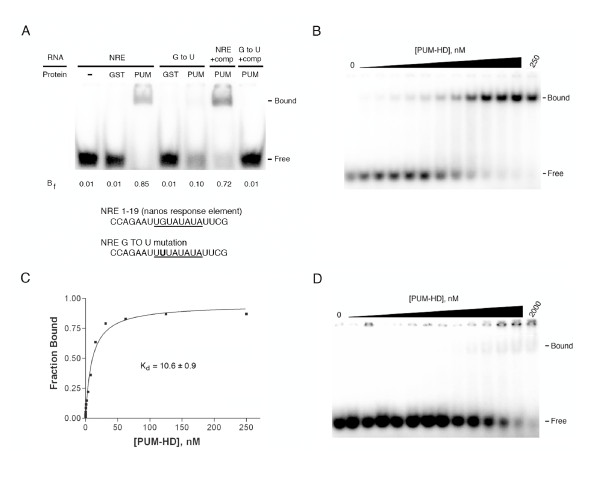
**AtPUM-HD-2 demonstrates binding specificity to wildtype NRE1**. EMSAs using purified GST-AtPum2 PUM-HD and wildtype or point mutated NRE1. (A) EMSA assays using the wildtype (NRE) or mutant (G to U) NRE1 RNA oligonucleotide in the absence or presence of GST or GST-AtPum2 PUM-HD (PUM). Unbound radiolabelled RNA (Free) shifts to a high molecular weight complex when bound to GST-AtPum2 PUM-HD (Bound). The fraction of bound RNA (B_f_) was determined for each reaction. 100-fold excess non-labelled mutant NRE1 was added to the reaction containing labelled WT-NRE (NRE + comp). Conversely, 100-fold excess of non-labelled wildtype NRE1 was added to the reaction containing labelled mutant NRE RNA (G to U + comp). The underlined RNA sequence corresponds to cognate RNA that interacts with repeats 1 through 8 in the Drosophila Pumilio PUM-HD. (B) EMSA titration of WT-NRE1 and increasing concentrations of GST-AtPum2 PUM-HD (PUM-HD). The protein concentrations were 0, 0.12, 0.25, 0.5, 1.0, 2.0, 3.9, 7.8, 15.6, 31.3, 62.5, 125 and 250 nM. (C) The fraction of bound WT-RNA as a function of GST-AtPum2 PUM-HD from the EMSA in (B) was plotted and the dissociation constant (K_d_) was determined. (D) EMSA titration of mutant NRE1 (G to U) and increasing concentrations of GST-AtPum2 PUM-HD. The protein concentrations were 0, 0.5, 1.0, 2.0, 3.9, 7.8, 15.6, 31.3, 62.5, 125, 250, 500, 1000 and 2000 nM.

To determine if the AtPum2 PUM-HD binds RNA as is predicted by structural modeling, electrophoresis mobility shift assays (EMSAs) were performed. Two synthetic 19-nucleotide RNAs were used in these assays. The first was a wildtype Nanos Response Element (wildtype NRE1) that matched a region from BoxB of the *hunchback *NRE1 (Figure 7A). This RNA oligonucleotide (wildtype NRE1) was identical in sequence to one used in a previous study that analyzed the binding affinity of the human PUM-HD to RNA [14]. The second RNA oligonucleotide was a variant form of the NRE1 (mutant NRE1) that contained a single nucleotide change in the highly conserved core of the Puf repeat binding site (UGU to UUU). This mutant NRE1 was shown to have approximately 100-fold reduced affinity for the human PUM-HD [14]. The EMSA experiments demonstrated that the AtPum2 PUM-HD bound effectively to the wildtype NRE1, whereas binding to the mutant NRE1 was significantly lower (Figure 7A). Competition assays were performed to further demonstrate the specificity of the AtPUM2 PUM-HD interaction with wildtype NRE1. The addition of 100-fold excess concentration of cold mutant NRE1 competitor to the assay mixture only slightly reduced the binding of wildtype NRE1 to the AtPum2 Pum-HD, whereas the addition of excess cold wildtype NRE1 competitor completely eliminated any detectable interaction between the protein and the mutant NRE1 (Figure 7A).

EMSA titration experiments were conducted to determine the binding affinity of the AtPum2 PUM-HD to the wildtype and mutant NRE1. The AtPum2 PUM-HD bound to wildtype NRE with an apparent dissociation constant of 10.6 nM (Figure 7B, 7C). This value is >10-fold higher than was observed for Drosophila Pumilio PUM-HD binding to the NRE1 (K_d_~0.5 nM; [34]), but within the range observed for other Puf protein interactions with their cognate RNAs [21,28]. The binding affinity of the protein to the mutant NRE1 was significantly lower than to wildtype RNA (Figure 7D). Although a protein-mutant NRE1 complex was apparent at an AtPum2 PUM-HD concentration of 62.5 nM, the interaction remained weak at a protein concentration of 2000 nM, as seen by the diffuse nature of the shifted band. However, only a small amount of free RNA was present in the 1000 nM and 2000 nM samples, indicating that the low affinity binding of the AtPum-HD to the RNA resulted in a dissociation of the complex during electrophoresis. Thus, the instability of the complex did not allow for an accurate determination of the dissociation constant. However, based on the amount of free RNA in each lane, the dissociation constant value for the protein bound to mutant NRE1 appears in the range of 250 to 500 nM.

### Arabidopsis Puf proteins typically localize to dynamic, punctate cytoplasmic structures

**Figure 8:**
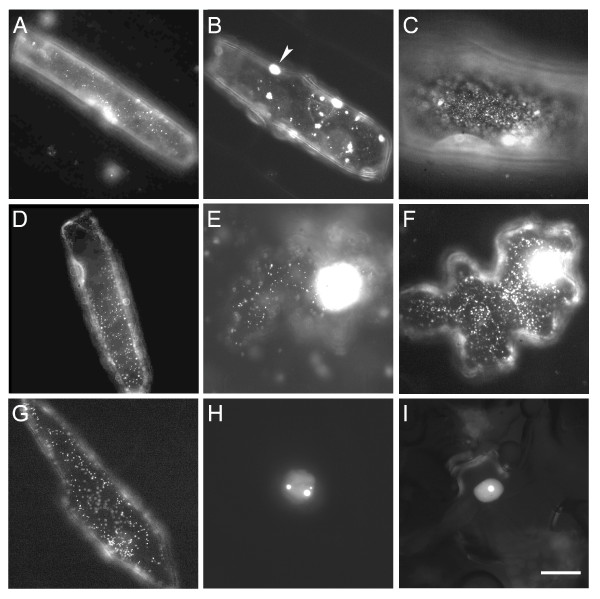
**Subcellular localization of AtPum proteins**. Representative epifluorescence images of cells expressing AtPum proteins fused to the amino terminus of GFP or RFP in onion (A to D) or fava bean (E to I) epidermal cells. (A) AtPum7-GFP, (B) AtPum8-RFP, (C) AtPum9-RFP, (D) AtPum10-GFP, (E) AtPum12-GFP, (F) AtPum14-GFP, (G) AtPum18-RFP, (H) AtPum23-GFP, and (I) AtPum24-GFP. Arrow identifies heavily stained region that likely resulted from aggregation of protein complexes. Bar, 10 microns.

**Figure 9:**
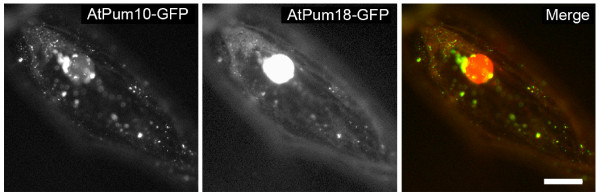
**Leptomycin B treatment results in an enrichment of AtPum18-RFP, but not AtPum10-GFP, in the nucleus**. Bar, 10 microns.

To provide insight into the subcellular localization patterns of the Arabidopsis Puf proteins, several of these proteins were transiently expressed as fusions to the amino-terminus of either GFP or RFP in onion or fava bean epidermal cells. In total, nine AtPum proteins were successfully expressed as fluorescent protein fusions. Seven of the fusion proteins localized to cytoplasmic structures that were visible as puncta 0.5 microns or less in diameter (Figure 8A to 8G). However, large protein aggregates were occasionally observed in some cells, likely resulting from higher levels of expression of the fusion protein (Figure 8B). In epidermal cells that demonstrated active cytoplasmic streaming, the fluorescent particles were occasionally dynamic, demonstrating stop-and-go movements that reached peak velocities of up to five microns/second (Additional file 4). Particle movement ceased when cells were treated with the actin destabilizing agent, latrunculin B (Additional file 5), indicating that the actin cytoskeleton was responsible for driving particle movement. The microtubule disrupting agent, oryzalin, did not noticeably affect the movement of the AtPum containing particles (data not shown). In addition to punctate cytoplasmic staining, strong nuclear fluorescence was often observed, and was more prevalent in cells expressing specific fluorescent protein fusions (e.g. Figure 8E and 8F). This observation suggested that some fusion proteins shuttle between the nucleus and cytoplasm. Nuclear localization and export may be common features for most of the AtPum proteins, as 24 out of the 26 proteins have a predicted leucine-rich nuclear export signal (NES) http://www.cbs.dtu.dk/services/NetNES/, with AtPum1 and AtPum4 as exceptions. The predicted NES sequences are located in the variable amino terminal region of each protein, which in several AtPum proteins is extremely short (Figure 2). NES-containing proteins are dependent on CRM1 (exportin 1) for export from the nucleus [35], and are sensitive to export inhibition by leptomycin B. Epidermal cells expressing each of the cytoplasmic, punctate AtPum fluorescent protein fusions shown in Figures 9A through 9G were treated with leptomycin B. We observed that all but one fusion protein (AtPum10-GFP) demonstrated an accumulation of fluorescent signal in the nucleus after treatment with leptomycin B (Figure 9, data not shown). This indicated that AtPum proteins shuttle between the cytoplasm and the nucleus via a CRM-1 mediated pathway.

**Figure 10:**
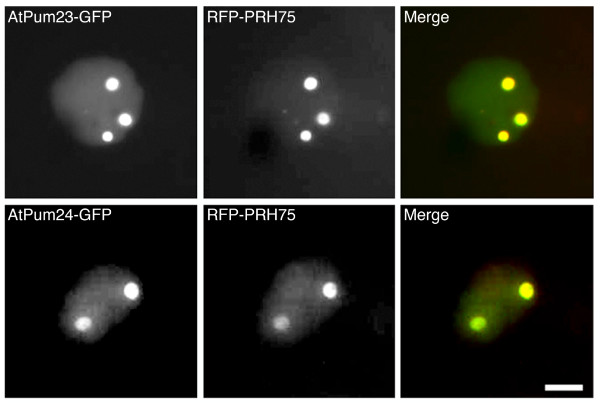
**AtPum23 and AtPum24 co-localize with the nucleolar marker RFP-PRH75**. Bar, 1 micron.

Two AtPum-fluorescent fusion proteins (AtPum23-GFP and AtPum24-GFP) had a different subcellular localization pattern from the others, demonstrating only nuclear fluorescence that was enriched within nucleolar-like structures (Figure 8H, I). Co-expression of AtPum23-GFP and AtPum24-GFP with a nucleolar targeted marker protein (RFP-PRH75; [36]) confirmed that these structures were indeed nucleoli (Figure 10). Interestingly, AtPum23 and AtPum24 are the only Arabidopsis Puf proteins with long polypeptide extensions at the carboxyl-terminal end of the PUM-HD. Nucleolar localization signals are not easily predictable; however, these signals are often enriched in the basic amino acids lysine and arginine, two amino acids that are well represented in the carboxyl-terminal regions of AtPum23 and AtPum24. These two proteins have predicted nuclear localization signals (NLS; Predict NLS, http://www.predictprotein.org/cgi/var/nair/resonline.pl). AtPum23 possesses a predicted NLS at position 688 near the carboxyl-terminal end of the protein, and AtPum24 has two predicted NLS sequences near the amino-terminal end (positions 77 and 98).

To determine whether the various AtPum proteins were segregated into distinct cytoplasmic particles, several pairs of AtPum-fluorescent protein fusions (AtPum7-GFP:AtPum18-RFP, AtPum9-GFP:AtPum18-RFP, AtPum12-GFP:AtPum18-RFP, AtPum14-GFP:AtPum18-RFP, AtPum10-GFP:AtPum8-RFP) were co-expressed in epidermal cells. Each pair of co-expressed AtPum fusion proteins co-localized within the same cytoplasmic particles (data not shown). However, there was a frequent concentration bias of either GFP or RFP fluorescence in co-localizing particles, indicating that AtPum proteins were not necessarily represented in equimolar concentrations within each particle.

## Discussion

The Arabidopsis and rice Puf gene families are extensive, consisting of a greater number of members than any other model species studied to date [9]. Considering the size of the Arabidopsis and rice genomes (Arabidopsis, 125 Mb, 26751 genes; rice, 389 Mb, 42,000 genes; [37-39]), Puf genes are over-represented in the plant genome when compared to other species. Whole genome duplications may have contributed to the large number of plant Puf genes. The ancestral Arabidopsis genome was duplicated three times in the past 150-200 million years [40], and the rice genome, along with that of other monocots, has probably experienced at least one duplication event [41]. In addition to whole-genome duplications, Puf gene expansion likely increased as a result of single gene duplications in both Arabidopsis and rice, as demonstrated by the presence of tandem gene copies (Figure 1). The presence of plant, algal and yeast sequences in the two main branches of the phylogenetic tree (Groups I and II, Figure 1) indicates that the PUM-HD of these proteins has remained relatively conserved in these species. Branches containing Arabidopsis sequences only (Groups III and IV) suggests that independent radiation of Puf proteins has occurred in this species, in a similar fashion to a large group of Puf proteins in *Caenorhabditis elegans *[9]. The maintenance of duplicated plant Puf genes may be related to the sessile lifestyle of plants and their ability to adapt to challenging environmental conditions. If plant Puf proteins function in mRNA decay and translational repression as they do in other organisms, they could function to regulate the stability or translation of their target mRNAs in response to environmental stimuli in a rapid and coordinated manner. Indeed, microarray profiling revealed extensive changes in AtPum transcript expression patterns in response to various external stimuli (Table 1).

Homology modeling of AtPum2, AtPum13 and Os01g62650 PUM-HDs predicted that these domains adopt the characteristic crescent shaped structure that is common to PUM-HDs (Figure 5, 6). The identity of the amino acids at positions 12, 13 and 16 in each of the AtPum2 and Os01g62650 Puf repeats are identical to those in the Drosophila Pumilio and human Pum1 proteins, and provide conserved interactions with their corresponding RNA bases in the NRE1 sequence (Figure 5; [6,12]). The predicted interaction between the AtPum2 PUM-HD and the NRE1 was confirmed by EMSA assays (Figure 7). The UGU core target sequence appears necessary for binding the AtPum2 PUM-HD, as demonstrated by the reduced affinity of AtPum2 to an NRE that contained a point mutation in this core sequence (Figure 7). This result is consistent with a recent study that utilized a three-hybrid assay to determine binding interactions [27]. The predictable association between amino acids within each Puf repeat and their bound nucleotide was revealed previously for human Pum1, where substitution of specific amino acid side chains demonstrated that an RNA recognition code exists for this protein [13,14]. However, Puf proteins appear able to interact with RNA binding sites that vary substantially in sequence outside of their UGU core. For instance, human Pum1 binds hundreds of mRNA targets *in vivo *[17,18], as do Puf proteins from Drosophila, *C. elegans *and yeast [15,16,28,42]. These subsets of mRNAs are typically related in function or in their subcellular localization pattern [15,16,18,24]. In addition to binding to RNA targets with sequence variability, the PUM-HD of several Puf proteins can accommodate binding targets that are greater than eight nucleotides in length by flipping out spacer nucleotides [19-21,43]. Evidence is emerging that individual plant Puf proteins also bind to a range of mRNA targets [27].

Many of the plant Puf proteins have considerable variability in the amino acids at position 12, 13 and 16 in their PUM-HD that are predicted to be involved in molecular interactions with RNA bases (Figure 4, Additional file 1). Several of these variable triplet amino acids are found in Puf proteins identified in other organisms [16,28], however, many are unique to plants. Should these variable Puf proteins indeed possess RNA binding activity, the amino acid sequence variability could provide another level of specificity for Puf mRNA targets in plant cells. The triplet amino acids in the Puf repeats of AtPum13 differ from those in AtPum2 in six of the eight Puf repeats (Figure 4), and modeling of AtPum13 indicated that the NRE1 is not an ideal target for this protein, based on the predicted absence of stacking interactions (Figure 6). The observation that several of the Arabidopsis and rice proteins have fewer than 8 recognizable Puf repeats might also provide a mechanism for variable RNA target specificity. Yeast Puf proteins that possess only six Puf repeats function as RNA-binding proteins that function in post-transcriptional control of gene expression [8,15]. Whether the plant Puf proteins that possess only two, three or four repeats are *bona fide *RNA-binding proteins or function in some other cellular capacity, remains to be determined. It is possible that one or more of these proteins are encoded by pseudogenes and are not functional. However, transcriptional array data indicates that these genes are actively transcribed in Arabidopsis (Table 1), providing support for their expression and activity.

Additional regions that were identified within Puf proteins could play a role in determining RNA target specificity. A histidine side chain in Puf repeat 8' from human PUM1 is involved in stacking interactions with the uracil base bound to Puf repeat 8 [12]. Repeat 8' is present in most plant PUM-HDs (Figure 2), and the modeled AtPum2 structure indicates that this conserved histidine does indeed provide a stacking interaction with the corresponding RNA base (Figure 5). The NABP domain located in the amino terminal region of several Arabidopsis and rice Puf proteins, and the additional Puf repeats in the AtPum23 amino terminal region (Figure 2), could also enhance specificity of RNA targets by binding to regions of the transcript that lie outside of the PUM-HD binding site. The recruitment of other factors might also enhance the RNA binding specificity of the Pum-HD. The convex surface of repeats 7, 8 and 8' in the Drosophila PUM-HD interacts with its co-factors Nanos and Brat, and this interaction involves an extended loop that lies between repeats 7 and 8 [6,33]. A conserved extended loop in the AtPum2 PUM-HD indicates that a similar interaction might also occur (Figure 5), although homologs of Nanos and Brat have not been identified in plants. Interestingly, the model of the AtPum13 PUM-HD structure reveals potential loops between Puf repeats 2 and 3, and repeats 3 and 4 (Figure 6). These loops also present potential binding surfaces for regulatory proteins.

Most of the AtPuf proteins that were expressed in epidermal cells as fluorescent protein fusions were localized to dynamic, punctate structures in the cytoplasm (Figure 8). The prevalence of predicted NES sequences in these proteins, and the observed nuclear accumulation of many of these after treatment with LMB indicates that nucleocytoplasmic shuttling is a common feature of these proteins. The enrichment of AtPum23 and AtPum24 fluorescent protein fusions within nucleoli provides another association of Puf proteins with the nucleus (Figure 8H, 8I). Nucleoli are traditionally known to be involved in the transcription and processing of ribosomal RNA and ribosome subunit biogenesis, and in the assembly of RNPs [44]. More recently, the discovery that numerous mRNAs, the exon-junction complex of proteins, RNA-binding proteins, and other proteins localize to nucleoli in plant cells supports a role for the nucleolus in mRNA processing, silencing, surveillance and export [44,45]. Additionally, a search of the human nucleolar database http://www.lamondlab.com/NOPdb3.0/ identified a human Pumilio-domain containing protein. As well, yeast Puf6p, an Ash1 mRNA-binding protein, is a nuclear shuttling protein that is enriched in the nucleolus [46,47], as is mammalian Staufen, another protein involved in cytoplasmic mRNA localization [48]. Thus, the nuclear associations of AtPum proteins suggest that these proteins are components of a preassembly complex that is involved in RNA decay, translational control or cytoplasmic transport of specific groups of mRNAs.

## Conclusions

The Puf family of RNA-binding proteins in Arabidopsis and rice contain a greater number of members than in any other model species studied thus far. The modeled three-dimensional structure of three plant PUM-HDs is conserved, however, the identity of the amino acids that are predicted to contact RNA bases demonstrates considerable variability throughout this family of proteins. EMSA and subcellular localization studies indicate that these proteins are nucleocytoplasmic shuttling proteins that bind to RNA in a sequence specific manner. The large number of plant Puf protein family members suggests that these proteins are key in regulating the stability and translation of a significant number of mRNAs in the cell. Important future studies include the identification of target mRNAs for individual plant Puf proteins, determination of co-crystal structures of divergent Puf proteins with their cognate RNAs, and identification of the components of Puf protein-containing particles.

## Methods

### Bioinformatic analysis of plant Puf genes

Basic Local Alignment Search Tool (BLAST) analyses were performed to identify Arabidopsis and rice genes that encode regions of the conserved PUM-HD. The amino acid sequence of the Drosophila PUM-HD (residues 1093 to 1427) [6] was queried against Arabidopsis (http://www.ncbi.nlm.nih.gov/genome/seq/BlastGen/BlastGen.cgi?taxid=3702; http://rarge.gsc.riken.jp/; http://www.tigr.org/tdb/e2k1/osa1/) rice http://rice.plantbiology.msu.edu/index.shtml, moss (*Physcomitrella patens*, http://www.cosmoss.org/), and *Chlamydomonas reinhardtii *http://www.chlamy.org/ sequence databases using BLASTp and tBLASTn programs.

Multiple sequence alignments of Arabidopsis and rice PUM-HDs were generated using ClustalX, and then manually refined in BioEdit (version 5.0.9; [49]). Ambiguously aligned sites were removed from the alignment, leaving 341 amino acids that were used in the phylogenetic analysis. Three types of analyses were performed; Bayesian inference, and two methods in the maximum likelihood framework (PhyML, RAxML). The Bayesian analysis was performed using MrBayes (v3.1.2) for 20,000,000 generations with trees sampled every 1000 generations [50,51]. The mixed model approach was implemented with MrBayes. This allowed new models to be proposed in the course of the analysis, with multiple models potentially contributing to the posterior distribution of trees. Maximum likelihood trees were estimated using the programs PhyML [52] and RAxML [53]. Model selection was guided by the Akaike Information Criterion as implemented in ProtTest [54]. In addition, 500 bootstrap replicates were performed using both PhyML and RAxML to assess statistical significance of the groups.

Transcript expression profiles are based on microarray data available from the public databases Genevestigator [31] and The Bio-Array Resource for Plant Functional Genomics (BAR) [32].

### Homology models

The sequences of the PUM-HD from AtPum2 (At2g29190, amino acids 616-952), AtPum13 (At5g43090, amino acids 252 to 527), and Os01g62650 (amino acids 707-1043) were aligned against the sequence of the PUM-HD from human PUM1 (Hs.281707, amino acids 828-1178, Protein database ID: 1M8X). Homology models were generated by Modeller 9v2 [55] using as a template the crystal structure of the PUM-HD from human Pum1 in a complex with a 14-mer RNA sequence from the NRE1-14 of the Drosophila *hunchback *mRNA (PDB code 1M8X, [12]. The homology models were refined with CNS 1.1 [56] and the quality of geometric parameters was assessed with PROCHECK [57]. All backbone torsion angles were within allowed regions of the Ramachandran plot, and more than 90% of the residues were in the energetically most favored regions. Figures were prepared using PyMOL software (DeLano Scientific).

### Molecular cloning

PCR was used to amplify the coding regions of several Arabidopsis Puf genes. Full-length complimentary (cDNA) or genomic DNA was used as a template for PCR. Full-length cDNA clones were obtained from RIKEN and the Arabidopsis Biological Resource Center (ABRC). For recombinant protein expression, the coding region of repeats 1' through 8' of the AtPum2 PUM-HD (amino acids 614 to 963) was amplified by PCR using oligonucleotide primers 5'CGAGGAGGATCCTTTGGATCTTCAATGCTTGAAG3' and 5'CGAGGAGGATCCGGCCATGTTGTAGAGTTCAGTTC3'. The PCR product was cloned into the BamHI and SalI sites of pGEX-6P-1 (GE Healthcare).

Construction of expression vectors for AtPum subcellular localization analysis was performed using recombination or ligation based cloning of full-length AtPum coding regions into various expression vectors. Oligonucleotide primers were designed to encode the full-length AtPum protein. The PCR primers used were: AAGATGGATGAGTTTCGTGAAG and CTTCTTCAATAGATTCCTCGAGAAA (AtPum7); CACCATGATGAGAGGTGAATTTGG and ATTCTTCAAGAGATTTCGTG (AtPum8); CACCATGGGTTTTGGAGGTTTTAATG and CTTCTTCAAGATGGTCTTG (AtPum9); TGCATGGAGATTTTTAACTTCGGAC and CTTCTTCAAGATGGTCTTGGAGAAAATC (AtPum10); GAGATGGATCAGAGAAGAGGAAATG and CTTCTTCGAGCTAAGTGCGGAGAGG (AtPum12); ACCATGGACAAGAATTTTCGTG and GATATTGAGTTTCTCCAGAACTTTG (AtPum14); CACCATGGCAGTCGCTGATAATCCC and GCAACGAAGCCTAATGAGTCCAAG (AtPum18); CACCATGGTTTCTGTTGGTTCTAAATCAT and AATTCTCATTTTATTTGAATGCCGA (AtPum23); CACCATGTCTTCCAAAGGTCTGAAACCTC and TTCAGGTTTCTTGGTTGCTGAGATC (AtPum24). For recombination cloning, PCR amplified products were inserted into the pDONR221 or pENTR/D-TOPO Gateway entry vectors (Invitrogen), which were then recombined with pB7FWG2 or p2GWF7 GFP fusion destination vectors (Functional Genomics Unit, Department of Plant Systems Biology, VIB-Ghent University). Ligation cloning of PCR products into the BglII and XbaI sites of pRTL2ΔNS/RFP [58] was performed for RFP fusion constructs. The expression vectors used the cauliflower mosaic virus (CaMV) 35S promoter to drive transcription, and either the 35S or nopaline synthase terminator sequences. The nucleotide sequence was confirmed in each expression construct by standard sequencing techniques (Quintara Biosciences, Berkeley, CA)

### Electrophoretic mobility shift assays (EMSA)

The AtPum2 PUM-HD coding sequence in pGEX-6P-1 was expressed in *E. coli *strain BL21(DE3) as a fusion to the carboxyl-terminus of glutathione S-transferase (GST). Bacterial cultures were grown overnight at 37°C, diluted 1 in 100 in fresh media and grown at 37°C to an OD_600 _value of 0.6. IPTG (0.2 mM) was added and the cultures were incubated at 37°C for 3 hours. The recombinant protein was purified using a glutathione affinity matrix (Stratagene), and reconstituted in binding buffer (10 mM HEPES pH 7.4, 1 mM EDTA, 50 mM KCl, 1 mM DTT, 0.01% BSA, 0.01% Tween 20). Synthetic RNAs (Dharmacon) were radiolabelled using ^32^P-γ-ATP (3000 Ci/mmol, Perkin Elmer) and T4 polynucleotide kinase (Fermentas). Labelled RNAs were purified from free nucleotides by gel filtration chromatography (NucTrap, Stratagene). Binding reactions (30 μL volume in binding buffer) contained 200 pM of labelled RNA and varying concentrations of protein, with or without cold competitor RNA. Reactions were incubated at room temperature for one hour, and run on a 6% non-denaturing acrylamide gel (Mini Protean II, BioRad) at 100 V for 45 minutes at 4°C. Gels were dried, exposed to a storage phosphor screen for 6-12 hours, and the screens scanned using a phosphorimager (Molecular Imager FX, BioRad). Densitometry was performed using Quantity One software (version 4.5.1, BioRad), and the data was analyzed using Prism 3 software (GraphPad). To determine the fraction of protein that was bound to RNA (fraction bound, B_f_), the relative pixel intensity in the bound complex band was divided by the sum of the pixel intensities in the bound complex band plus the free RNA band.

### Transient expression in leaf epidermal cells and microscopic analysis

GFP and RFP fusions proteins were expressed in fava bean (*Vicia faba*) and onion (*Allium cepa*) epidermal cells layers using the particle bombardment technique (PDS-1000, BioRad) [59]. After bombardment, leaves were incubated overnight in the dark. Epidermal cell layers were peeled from the leaves, mounted on microscope slides in distilled water, and covered with a coverglass. For drug treatments, onion epidermal cell layers were floated on a Murashige and Skoog (MS) medium containing either leptomycin B (190 ng/mL, Sigma) and latrunculin B (1 nM, BIOMOL International). Negative control peels were floated on MS medium containing an appropriate volume of the drug solvent only. Epidermal cell layers were observed through FITC or rhodamine filter sets using a Plan Fluotar 40x objective lens or a Plan Apo 63x oil immersion objective lens attached to an epifluorescence microscope (DMR, Leica). Images were captured using a cooled CCD camera (Retiga 1350 EX; QImaging). Velocity software (Version 4.3.1, Improvision) was used for capturing images and image series compilation. Adobe Photoshop software (Version 8.0, Adobe Systems Inc.) was used for image modification and assembly.

## Abbreviations

At: *Arabidopsis thaliana*; BLAST: Basic Local Alignment Search Tool; Brat: Brain Tumor; DUF: domain of unknown function; EMSA: electrophoresis mobility shift assays; FBF: *fem-3 *binding factor; hb: hunchback; GFP: green fluorescent protein; NABP: nucleic acid binding protein; NES: nuclear export signal; NLS: nuclear localization signal; NOS: Nanos; NRE: Nanos response element; Open reading frame (ORF); Os: *Oryza sativa*; PUM-HD: Pumilio homology domain; RFP: red fluorescent protein; UTR: untranslated region.

## Authors' contributions

DGM and PPCT designed the experiments. PPCT, IHB-N and MWCT conducted the modeling analysis. PPCT, DMS and MWCT performed the bioinformatics analysis. PPCT, DGM and ALA performed the cell biology studies. DGM conducted the EMSA assays. DGM and PPCT wrote the manuscript. All authors read and approved the final manuscript.

## Supplementary Material

Additional file 1 **Supplemental Figure 1 - Amino acid sequence alignment of the PUM-HDs of all *Arabidopsis thaliana*, *Oryza sativa*, *Physcomitrella patens*, *Chlamydomonas reinhardii*, *Homo sapiens*, *Drosophila melanogaster*, and *Saccharomyces cerevisiae *Puf proteins**. Residues shaded in black indicate amino acid identity and residues shaded in grey indicate amino acid similarity. Amino acid residues are shaded when greater than 60% of the amino acids are conserved at that position. Click here for file

Additional file 2 **Supplemental Table 1 - Pair-wise comparative sequence analysis of the amino acids located in the amino-terminal extensions that lie outside of the PUM-HD in AtPum proteins**. A comparison of the percentage of amino acid identity and similarity for the amino terminal extensions from each protein are shown (Identity/Similarity). Click here for file

Additional file 3 **Supplemental Figure 2 - Stereo image of the ribbon structure of AtPum2 shown in Figure **5. Click here for file

Additional file 4 **Supplemental Movie 1 - Cytoplasmic Arabidopsis Pum particles are dynamic**. AtPum18-RFP was expressed in onion epidermal cells and imaged at 4 frames per second and displayed in real time. The intensely staining nucleus is visible at the top of the image. Click here for file

Additional file 5 Supplemental Movie 2 - Latrunculin B disruption of actin filaments results in arrested movement of AtPum18 particles. Click here for file
