# Supplementary figures and images for: The Puf family of RNA-binding proteins in plants: phylogeny, structural modeling, activity and subcellular localization
Source: BMC Plant Biol. 2010 Mar 9;10:44. doi: 10.1186/1471-2229-10-44 (PMC2848763; doi:10.1186/1471-2229-10-44)

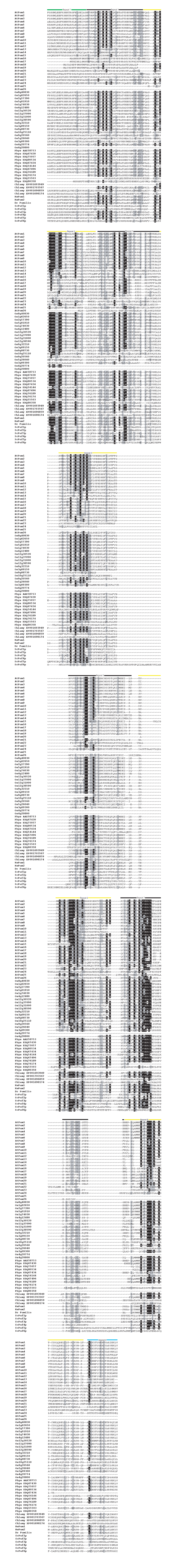

Supplement: Additional file 1 — Supplemental Figure 1 - Amino acid sequence alignment of the PUM-HDs of all Arabidopsis thaliana, Oryza sativa, Physcomitrella patens, Chlamydomonas reinhardii, Homo sapiens, Drosophila melanogaster, and Saccharomyces cerevisiae Puf proteins. Residues shaded in black indicate amino acid identity and residues shaded in grey indicate amino acid similarity. Amino acid residues are shaded when greater than 60% of the amino acids are conserved at that position. [file 1471-2229-10-44-S1.JPEG]

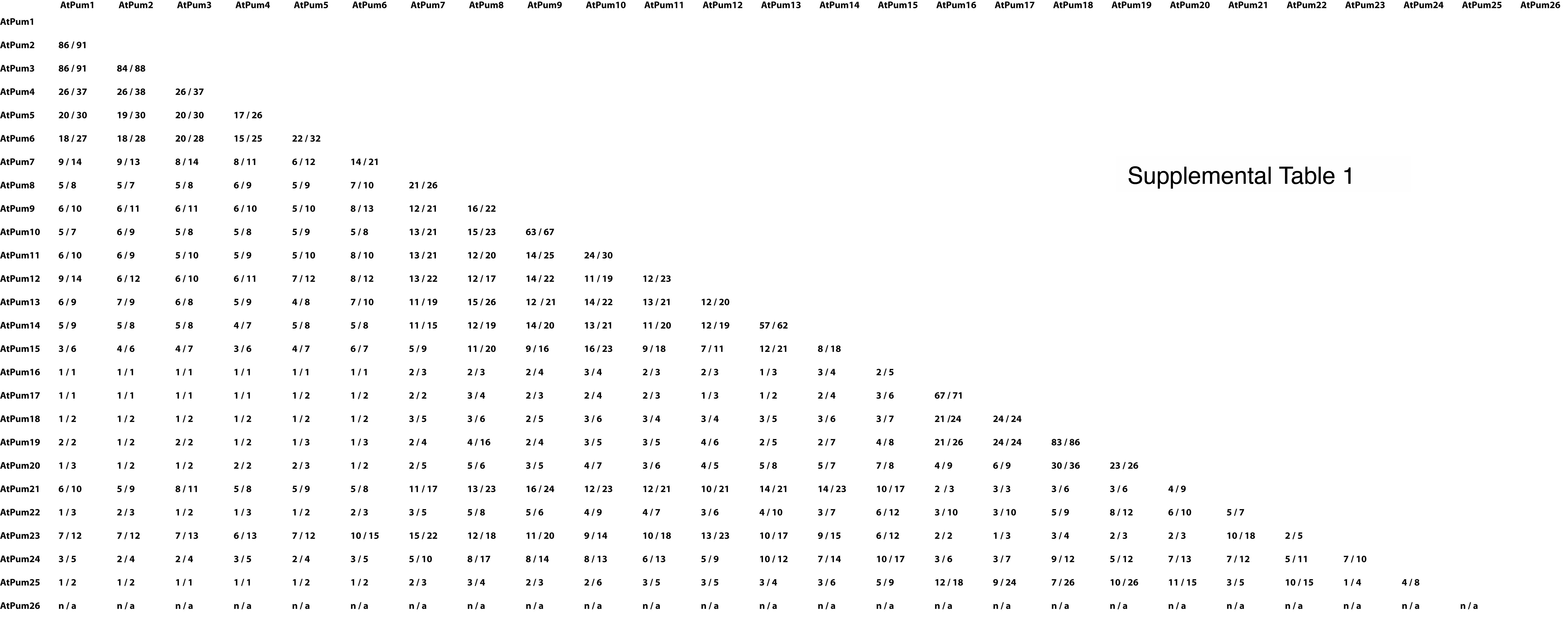

Supplement: Additional file 2 — Supplemental Table 1 - Pair-wise comparative sequence analysis of the amino acids located in the amino-terminal extensions that lie outside of the PUM-HD in AtPum proteins. A comparison of the percentage of amino acid identity and similarity for the amino terminal extensions from each protein are shown (Identity/Similarity). [file 1471-2229-10-44-S2.JPEG]

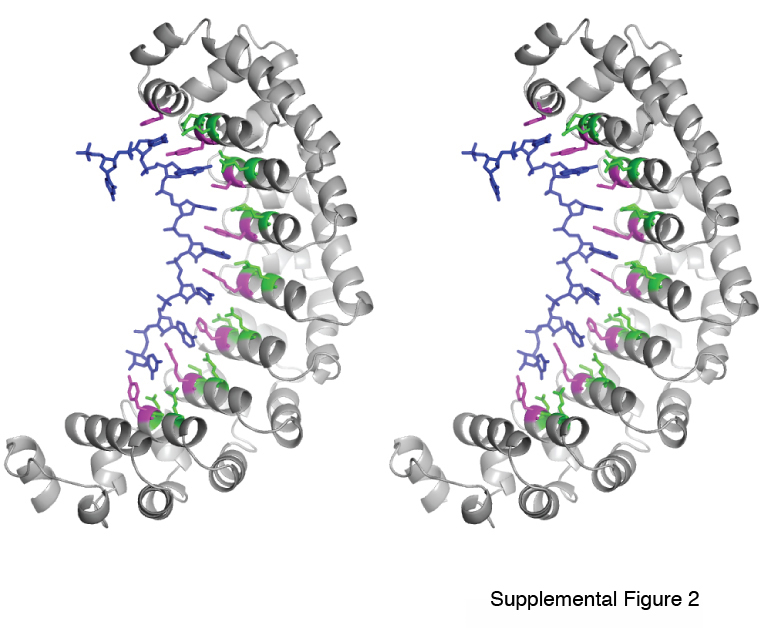

Supplement: Additional file 3 — Supplemental Figure 2 - Stereo image of the ribbon structure of AtPum2 shown in Figure 5. [file 1471-2229-10-44-S3.JPEG]
